# Evaluating multimodal commercial and open-source large language models for dynamical astronomy: a benchmark study of resonant behavior classification

**DOI:** 10.1038/s41598-026-45926-y

**Published:** 2026-03-28

**Authors:** Evgeny Smirnov, Valerio Carruba

**Affiliations:** 1 Belgrade Astronomical Observatory, Volgina 7, Belgrade, Serbia; 2 Department of Mathematics, São Paulo State University (UNESP), 333, Av. Dr. Ariberto Pereira da Cunha, Guaratinguetá, SP 12516-410 Brazil; 3 Laboratório Interinstitucional de e-Astronomia, Rio de Janeiro, RJ 20765-000 Brazil

**Keywords:** Engineering, Mathematics and computing

## Abstract

We present a systematic evaluation of modern multimodal large language models (LLMs) for the classification of mean-motion and secular resonances from images of resonant arguments. Four benchmark datasets (RB-TEST, RB-PILOT, RB-SMALL, RB-FULL) were constructed to cover clear, ambiguous, and transient cases, with both binary and three-class outputs. Using standardized prompts (a full prompt for large models and a simplified variant for small models that cannot process complex instructions), we tested flagship commercial models, large open-source models, and small locally runnable models. Commercial LLMs reach $F_1=100\%$ on simple cases and up to $94\%$ on the three-class RB-SMALL dataset, while the best open-source models also reach $100\%$ on unambiguous cases and $76\%$ on the complex ones. On the full binary benchmark, open-source models approach commercial performance ($F_1\approx 90$–$96\%$). Most errors occur in transient and resonance-sticking regimes. The results show that LLMs can perform resonance classification at levels comparable to those of classical or machine-learning methods without training or fine-tuning, and that even small open-source models achieve practically useful accuracy. The released benchmarks establish a reproducible standard for evaluating LLMs on dynamical astronomy tasks.

## Introduction

Machine learning has been used in astronomy for many years. Classical methods such as k-nearest neighbors, decision trees, random forests, or gradient boosting have helped classify images, detect outliers, and identify resonant asteroids^1,2^. Neural networks and deep-learning models have also been applied to resonance identification, asteroid family detection, and image analysis^2–4^. These methods demonstrated acceptable performance on many tasks. Under controlled conditions, when the integrator, time step, output frequency, the region of space, and other relevant parameters are all fixed, and a sufficiently large training set is available, classical machine-learning methods can achieve very high accuracy. Even simple classifiers such as decision trees may outperform in such cases^5^.

However, these models are tightly coupled to the specific conditions under which they were trained. When any of these parameters change (a different integrator, a different output frequency, or a different region), performance can degrade substantially^2,5^. The same applies to complex dynamical regimes. In regions of phase space where multiple resonances overlap and the resonant argument, which represents the linear combination of mean frequencies of a small body and planet(s), exhibits irregular, noisy, or mixed behavior, no single set of features or feature combinations provides a reliable basis for classification, which is essential for supervised models.

More fundamentally, all supervised machine-learning methods share an essential limitation: they require a training dataset specifically prepared for the given task. As a result, each new classification problem demands its own training dataset, adaptation of the architecture, hyperparameter tuning, and extensive computational resources. A model trained to detect a particular mean-motion resonance (MMR) cannot automatically identify a different resonance. Similarly, a model built to classify secular resonances cannot be reused for mean-motion cases without additional training or, what is more frequent, creating its own model^2^. Recent work on co-orbital dynamics has shown that unsupervised methods, such as statistical sparse jump models, can identify transitions between different regimes of the 1:1 mean-motion resonance without labeled training data, although the approach is tailored to a specific resonance type^6^. Scaling such an approach to the full diversity of resonances encountered in population-level studies, where hundreds of candidate resonant arguments per object may need to be inspected^7,8^, would require either an impractically large number of specialized models or an ensemble system whose complexity and maintenance cost would likely exceed the benefit. This lack of universality creates a substantial barrier for astronomical problems where the patterns vary from resonance to resonance, and where labeled datasets are expensive to produce or may require human attention.

Beyond machine learning, the problem of resonance identification has been addressed by deterministic methods. The most widely used approach is the direct analysis of the resonant argument: one computes the time series of the resonant angle and determines whether it librates or circulates^9,10^. More recent geometric and frequency-based techniques, such as the FAIR method^11^, can identify mean-motion resonances directly from orbital data without requiring a priori specification of the resonance type or order. Phase–portrait analysis provides another possible option, although it is used less frequently in population-level studies. These deterministic methods perform well in several important regimes: when only a few resonant arguments need to be inspected, in model problems with limited perturbations, and in clean dynamical cases where an object is either clearly trapped in a resonance or clearly outside it. In such situations, simple algorithmic criteria, e.g., checking whether the unwrapped resonant argument remains bounded, can achieve near-perfect accuracy^12^.

However, both machine-learning and deterministic approaches encounter difficulties in the same regime: complex, ambiguous cases arising in densely populated regions of phase space. In the main asteroid belt, where numerous two-body and three-body mean-motion resonances (especially high-order ones), secular resonances, and von Zeipel–Lidov–Kozai resonances overlap, algorithmic classification of resonant arguments^12,13^ achieves high accuracy (95–$100\%$) for unambiguous libration and obvious non-resonant cases, but degrades substantially for transient captures, resonance sticking, and other borderline dynamical regimes. A similar conclusion was reached in the study of near-Sun asteroids, where an automated algorithm based on windowed linear fitting of resonant arguments was developed to identify mean-motion and secular resonances across a large synthetic population; despite achieving approximately $95\%$accuracy on a benchmark sample, the method still required careful handling of overlapping resonances and cases where no mechanism could be identified^14^. Machine-learning methods require retraining for each new resonance type and struggle with the same edge cases. It is precisely these complex situations (where traditional criteria are insufficient and where visual inspection by an expert has been the only reliable alternative) that motivate the present study.

Large language models (LLMs) offer an approach that addresses both limitations simultaneously. Unlike supervised machine-learning methods, LLMs do not require task-specific training data; unlike deterministic algorithms, they can handle the visual complexity of ambiguous resonant arguments. These models can operate in the zero-shot regime, meaning that classification does not require a task-specific training dataset. Instead, the researcher provides an instruction written in natural language, and the model performs the task based on its general reasoning abilities. In the earlier study^15^, the author demonstrated that the model gpt-4-vision-preview was able to classify mean-motion resonances from images of resonant arguments with $100\%$ accuracy on simple examples, without training or fine-tuning. That work showed that an LLM can identify libration and circulation directly from visual patterns if the prompt describes the task clearly. It also showed that, unlike classical ML, an LLM is not restricted to a single resonance or a single dataset.

However, the models available at that time were still limited. Only a few of them included vision capabilities, and even those early vision-enabled models could process images only in restricted formats or resolutions. In contrast, modern LLMs are fully multimodal. Current flagship commercial models (such as gemini-2.5, gpt-5, and claude-4.5-sonnet) are multimodal, accept images as input, and can analyze plots without preprocessing. At the same time, several strong open-source multimodal models have appeared, including llama3.2-vision, gemma3, mistral-3.2, and others. These models can be used locally, without relying on external APIs. Furthermore, some of them, such as gemma3-1b/4b, require only modest computational resources. As a result, it is now possible to evaluate resonant argument classification using a wide spectrum of LLMs, from the largest commercial models to small open-source models that can run on a personal computer.

The present work builds on these developments. Our goal is to perform a systematic evaluation of LLM performance in a real astronomical problem: the automatic analysis of the resonant argument of an object, which is an essential and, arguably, the most difficult stage required to identify of mean-motion and secular resonances. The inputs for large language models are images (and hence, only multimodal models accepting images as inputs are considered); the outputs are categories. Unlike previous LLM-related studies that focus on broad-language benchmarks, general multimodal benchmarks, or question-answering datasets^16–19^, we focus on a concrete dynamical classification task that traditionally requires human inspection. We develop and release a benchmark that includes clear, ambiguous, and mixed cases of resonant behavior, covering libration, circulation, chaotic evolution, resonance sticking, and transient capture. Importantly, the benchmark includes both mean-motion and secular resonances, combining these tasks into one universal dataset.

We evaluate a wide set of models: leading commercial LLMs, large open-source models, and small multimodal models that researchers can run on their laptops. The latter group is of particular interest for practical research. While flagship models achieve the highest accuracy, their use requires paid API access and becomes expensive when classifying thousands of images. In contrast, small open-source models can run locally on ordinary hardware and do not depend on external services, which is important for astronomers having limited access to financial resources. Understanding how well these models perform on a real scientific problem is, therefore, essential for researchers who need reproducible and cost-effective tools.

The main contribution of this study is the creation of a comprehensive, publicly available benchmark for resonance argument’s classification that can be used to (1) compare LLMs and (2) select the right tool for the concrete scientific research. Our results demonstrate the strengths and limitations of different model categories, show that LLMs can classify resonances without training, and provide a foundation for further research on LLM-assisted dynamical astronomy. The benchmark also enables reproducible comparisons between models, ensuring that future developments in multimodal LLMs can be assessed on a consistent scientific task.

In this work, we focus on the classification of the dynamical behavior of predefined resonant arguments, rather than on the discovery of resonances themselves. The proposed approach assumes that a candidate resonance and its associated resonant argument have already been identified through standard analytical or numerical techniques. This is a deliberate methodological choice that reflects the structure of the resonance identification pipeline. In practice, identifying whether an object is trapped in a resonance involves several steps (and they will be discussed in details later in this manuscript): numerical integration of the equations of motion over a sufficient time span, computation of the resonant argument, classification of its behavior (libration, circulation, or mixed dynamics), and, in some cases, verification through frequency analysis (e.g., comparing the dominant periods of the resonant argument and the semi-major axis for MMRs)^9,20^. Among these steps, numerical integration is a well-established and robust procedure, and frequency matching works reliably in most cases because libration of the resonant argument induces corresponding oscillations in orbital elements such as the semi-major axis.

The principal bottleneck lies in the classification of the resonant argument itself. For objects in clean resonances, simple geometric criteria are enough: for instance, checking whether the unwrapped resonant argument remains bounded within $2\pi$and has oscillations, or whether the number of full revolutions does not exceed one. However, in crowded regions of phase space, particularly in the main asteroid belt, the dynamics are far more complex. Resonance overlap, temporary captures, and resonance sticking produce noisy, ambiguous time series where traditional algorithmic criteria fail to provide reliable classification^9,12,21^. These are precisely the cases that motivate the use of large language models: not to accelerate the classification of trivial cases, but to automate the visual inspection of complex and ambiguous resonant arguments that would otherwise require expert judgment.

The practical importance of this automation becomes apparent when one considers the scale of the problem. For a single main-belt asteroid, the number of candidate resonances of sufficiently high order can reach hundreds, including two-body resonances with Mars, Jupiter, Saturn, and Earth, as well as three-body resonances involving all possible planetary combinations^7,8^. While a significant fraction of these can be filtered out by simple criteria, a substantial number of ambiguous cases remain, making manual inspection impractical for population-level studies. The role of LLMs in this context is therefore to replace or minimize the need for astronomer-level visual inspection at scale, enabling systematic resonance surveys that would otherwise be prohibitively labor-intensive.

## Methods

To investigate the applicability of large language models for astronomical classification tasks, we developed a comprehensive benchmark dataset specifically designed for the visual classification of resonant argument behavior in celestial mechanics. In other words, inputs are images and outputs are categories. This study presents the creation of four datasets: RB-TEST, RB-PILOT, RB-SMALL, and RB-FULL, which enable systematic evaluation of LLM performance on astronomical time-series classification tasks. All datasets assume that the resonance under consideration, its order, and the corresponding resonant argument are known a priori. The benchmark, therefore, evaluates the ability of LLMs to classify the dynamical behavior of resonant arguments (which is the only task requiring possible human attention), rather than to identify resonances in an automated or blind-search sense.

### Theoretical considerations

**Fig. 1:**
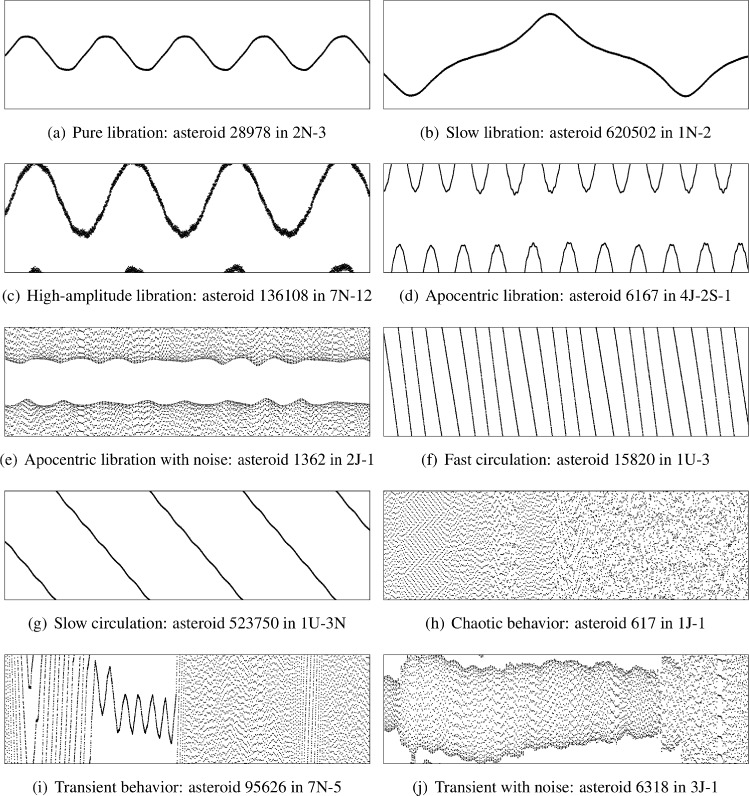
Representative examples of resonant argument behavior patterns from numerical integrations. The vertical axis displays the resonant argument ranging from 0 to 2$\pi$ radians, while the horizontal axis represents time in years. Each subplot demonstrates a distinct dynamical behavior utilized in the classification taxonomy, with characteristic timescales and amplitude variations visible in the phase-space representation. Asteroid designations and resonance identifications are provided for each case. The images are presented here in the same way as they are supplied to LLMs, without unnecessary details.

Although this research focuses on evaluating different large language models, it is essential to outline the scientific problem to which it applies. Mean-motion and secular resonances play an essential role in the dynamics of the Solar System^9^. A mean-motion resonance between a celestial body and a planet (or planets) occurs when there is a commensurability between the main frequencies of the involved bodies:

1 $$\begin{aligned} \sigma = \sum _{i=1}^{N} \left( \lambda _i m_i + \varpi _i p_i + \Omega _i q_i \right) , \end{aligned}$$

where $\sigma$ is the resonant angle (or resonant argument), *N* is the number of bodies involved ($N=2$ for two-body MMRs, $N=3$ for three-body MMRs), $\lambda _i$, $\varpi _i$, and $\Omega _i$ are the mean longitudes, longitudes of periapsis, and longitudes of ascending node of the bodies, respectively, and $m_i$, $p_i$, and $q_i$ are integers following the D’Alembert rule: $\sum _i (m_i + p_i + q_i) = 0$. The resonant angle may librate (oscillate), see Fig. 1(a), which indicates that the body is trapped in the mean-motion resonance; circulate (not librate), see Fig. 1(f), 1(g), 1(h), which indicates that the object is outside the resonance; or exhibit transient or mixed behavior, meaning that it is close to the separatrix or undergoing resonance sticking, see Fig. 1(i) and 1(j). Libration can occur near $\pi$ (e.g., Fig. 1(a)), near 0 or $2\pi$ (e.g., Fig. 1(d)), or around another center (e.g., Fig. 1(c)).

To identify whether an object is trapped in a mean-motion resonance, one can proceed as follows: (1) determine possible MMRs whose resonant semi-major axes lie near the object’s semi-major axis; (2) integrate the equations of motion of the system over a reasonable timespan (e.g., 100 kyr); (3) compute the corresponding resonant arguments; (4) determine whether these arguments librate; and (5) verify additional criteria, such as the closeness of the oscillation frequencies of the resonant angle and the semi-major axis. Detailed guides and software implementations are available in the literature: for two-body MMRs^9,20^; for three-body MMRs with Jupiter and Saturn^10,22^; for three-body MMRs for all planetary configurations^7^; software package resonances that identifies resonances automatically without the use of LLMs^12,13^. Technical details related to this study are provided in Subsection Dataset Construction

The principal challenge in this identification is the automatic classification of the resonant angle’s behavior: while a human expert may, in most cases, perform this task quickly and accurately (though in the most complex cases inter-rater reliability diminishes, arguing for a more robust and objective approach), the performance of automatic procedures remains limited, especially in transient cases^12,15^. This constitutes the task for the LLM: to act as a human observer, classify images of resonant arguments, and determine whether there is libration, circulation, or transient behavior.

A similar situation applies to secular resonances. They occur when there is a commensurability between the precession frequencies of the perihelion longitude (*g*) and the orbital node longitude (*s*) of an asteroid, and the fundamental frequencies of planetary theory, $g_i = \dot{\varpi }_i$ and $s_i = \dot{\Phi }_i$, where i is a suffix that identifies a planet in the Solar System (further details are discussed in the book)^23^. The frequencies associated with these resonances must obey the following relationship:

2 $$\begin{aligned} p\dot{g} + q\dot{s} + \sum (p_i\dot{g}_i + q_i \dot{s}_i ) = 0 , \end{aligned}$$

where the integers $p, q, p_i, q_i$ must satisfy D’Alembert rules. The equation 2 represents a secular resonance condition, where $\dot{g}$ and $\dot{s}$ are, respectively, the precession frequencies of the perihelion and the ascending node of the asteroid, while $\dot{g}_i$ and $\dot{s}_i$ are the equivalent frequencies of the planets. A linear secular resonance occurs when there is a simple commensurability between the perihelion precession frequency (*g*) or ascending node frequency (*s*) of an asteroid and the secular frequencies of a planet, such as $g_i$ or $s_i$^9^. Examples are the $g-g_6= {\nu }_6, s-s_6 = {\nu }_{16}$ secular resonances, among others.

Non-linear secular resonances are higher-order resonances whose argument can be written as combinations of arguments of linear resonances. An example is the $z_1 = g-g_6+s-s_6$ resonance, which can also be written as $z_1 = {\nu }_6+{\nu }_{16}$. Further information on secular resonances in the asteroid main belt can be found in the literature^24,25^. Additional discussion on the interaction of asteroid families with these resonances, including implications for their ejection-velocity fields, is also available^26^.

As an example of the dynamics of secular resonances, let us discuss the case of the ${\nu }_6$ linear secular resonance with Saturn. The dynamics inside the $\nu _6$ secular resonance are characterized by the evolution of the resonant argument,

$$\sigma _6 = \varpi - \varpi _6.$$

The resonant argument $\sigma _6$ here is the difference between the longitude of the asteroid’s perihelion ($\varpi$) and the corresponding longitude of Saturn ($\varpi _6$). The resonance itself corresponds to a one-to-one commensurability where the asteroid’s perihelion precession frequency (*g*) is approximately equal to Saturn’s frequency ($g_6$).

An asteroid within the resonance exhibits either circulation or libration of $\sigma _6$. In a circulating state, $\sigma _6$ varies continuously from $0^\circ$ to $360^\circ$. In a librating state, the argument oscillates, or is bounded, around an equilibrium point. Two equilibrium points are possible:

1. **Aligned Libration:** Oscillation around $0^{\circ }$. Here, the perihelia of the asteroid and Saturn are roughly oriented in the same direction.
2. **Anti-aligned Libration:** Oscillation around $180^{\circ }$. This state typically occurs when the asteroid’s orbit is closer to Saturn’s.

As a perihelion secular resonance, ${\nu }_6$ acts as a mechanism that pumps up the asteroid’s eccentricity while maintaining a relatively constant inclination. Consequently, a body evolving in this resonance may have its eccentricity increased indefinitely.

**Fig. 2:**
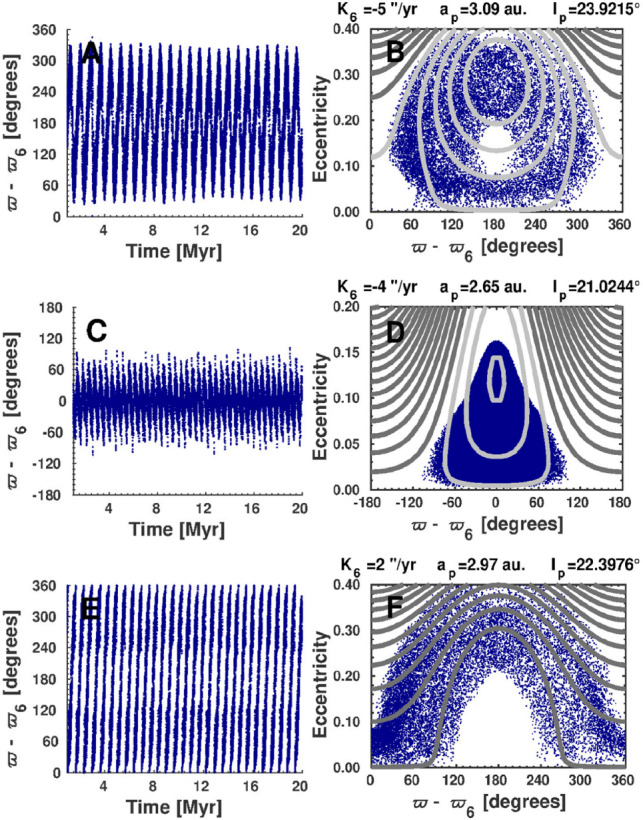
Three possible dynamical states for the ${\nu }_6$ resonance: anti-aligned libration, aligned libration, and circulation for asteroids (322770) 2001 FD145 (panel A), (337335) 2001 EQ18 (panel C), and (154204) 2002 HL8 (panel E). The evolution in the plane (${\varpi }-{\varpi }_6$, e) is depicted in Panels B, D, and F as blue dots that overlap the level curves of the analytical model of Yoshikawa (1987) for the corresponding value of $K_6$, the distance in frequency from the resonance center. Dark grey levels indicate the circulation domain, whilst light grey levels indicate the libration domains. $a_p$ and $i_p$are the asteroids’ synthetic proper elements, as reported from the AstDyS website. Reproduced from Fig. (3)^29^.

Anti-aligned orbits are actually circulating orbits trapped by a loop formed by the resonance separatrix. Because of the protection mechanism provided by this loop, anti-aligned orbits tend to be stable on timescales of several Myr^27^. Stable aligned orbits are rarer, since there are no protection mechanisms for these orbits. The few known asteroids in aligned orbits are located in the central main belt, in agreement with predictions from analytical models^28^. Figure (2) shows examples of real asteroids in anti-aligned libration, aligned libration, and circulation for the ${\nu }_6$ secular resonance.

The dynamics of non-linear secular resonances are similar to those of the ${\nu }_6$ resonance in terms of equilibrium points. Most asteroids interacting with non-linear secular resonances are on anti-aligned orbits, with rare exceptions in aligned configurations, as confirmed by recent works that attempted to identify asteroids interacting with non-linear secular resonances through deep learning methods^2^and Vision Transformers^4^.

### Dataset construction

Our benchmark construction methodology focused on two fundamental classes of resonances that govern the dynamics of small bodies in the Solar System: mean-motion resonances and secular resonances. Building upon extensive previous research on resonance identification^2,4,12,15^, we developed in this study a comprehensive classification scheme that captures the diverse phenomenology observed in resonant arguments through rigorous numerical integration and systematic categorization.

The datasets were constructed by systematically analyzing the visual patterns of resonant arguments over integration periods spanning $10^5$ years for MMRs and $>10^6$years for secular resonances, utilizing high-precision numerical integrators to ensure dynamical accuracy. We used the results obtained by different researchers in previous studies, including regular two-body MMRs^20^, three-body MMRs^1,10^, high-order two-body and three-body MMRs in the main belt^8^ and trans-Neptunian region^21^, and main belt secular resonances^2,4^. Some images have been created specifically for this study. Each resonant argument was classified into one of four primary categories based on its dynamical behavior:

1. Resonant: Objects whose resonant argument exhibits libration throughout the entire integration period. The resonant argument oscillates around a fixed equilibrium point, indicating capture in the resonance. The libration amplitude remains bounded within specific angular constraints.
2. Non-resonant: Objects displaying either chaotic behavior or circulation of the resonant argument. These objects are not trapped in the resonance and show no periodic oscillations. The resonant argument either monotonically increases/decreases or exhibits stochastic behavior.
3. Transient: Objects that alternate between periods of libration and circulation, indicating temporary capture in the resonance or resonance sticking, near-separatrix motion, followed by escape. These transitions must persist for a sufficient duration to distinguish from numerical artifacts.
4. Controversial: Cases where classification as transient or non-resonant depends on subjective interpretation and additional dynamical considerations. These edge cases represent the boundaries of classification confidence.

### Classification taxonomy

Based on the analysis and our previous experience, we introduced an extended taxonomy. Each primary category was further subdivided to capture the nuanced behavior observed in resonant dynamics through detailed phenomenological analysis. Figure 1 illustrates representative examples of almost every subtype, arranged to optimize visual comparison of distinct dynamical patterns observed in our numerical integrations.

**Fig. 3:**
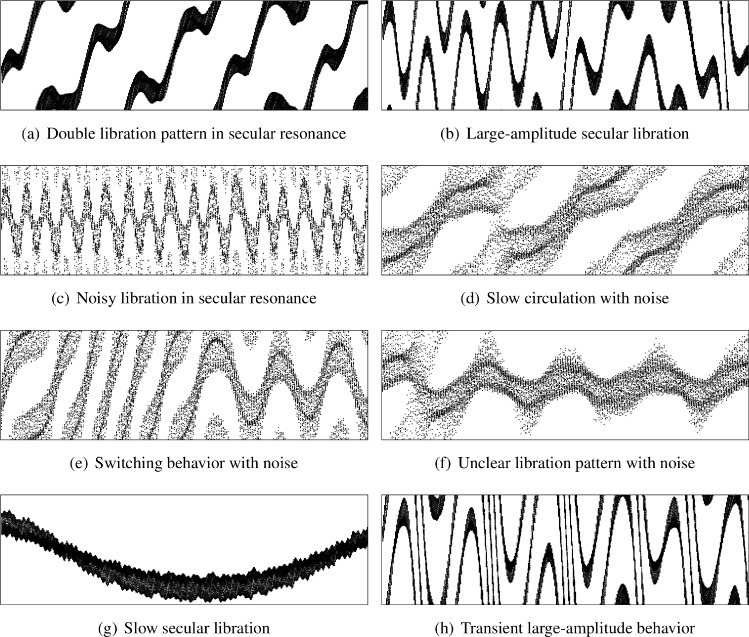
Representative examples of secular resonant argument behavior patterns from numerical integrations. The vertical axis displays the secular resonant argument ranging from 0 to 2$\pi$ radians, while the horizontal axis represents time in years. Each subplot demonstrates a distinct dynamical behavior in secular resonances, exhibiting characteristic timescales ranging from short-period oscillations to long-term evolutionary trends. The diversity of patterns reflects the complex interplay between secular perturbations and orbital dynamics.

Despite the apparent simplicity of librating behavior, our analysis identified several distinct patterns that significantly impact automated classification accuracy:

- Pure libration: Clear oscillations of the resonant argument are visible throughout the integration period, with at least 3–5 complete libration periods observable in the time series. The Fourier spectrum exhibits a dominant peak at the libration frequency (Fig. 1(a)).
- Slow libration: The libration period is comparable to the integration time, resulting in approximately one complete oscillation cycle being visible. This requires careful spectral analysis to distinguish from secular drift (Fig. 1(b)).
- Very slow libration: Only a fraction of a single libration period is captured within the integration timespan, characteristic of long-period resonances with small libration amplitudes. Extrapolation is necessary to confirm periodic behavior.
- High-amplitude libration: Libration with amplitudes approaching or even exceeding the critical values of 0 and 2$\pi$, creating complex visual patterns that may cross these boundaries (Fig. 1(c)).
- Double libration: Superposition of multiple harmonic components arising when an asteroid is simultaneously influenced by multiple resonances, such as a strong two-body resonance and a weaker three-body resonance or a secular resonance. Spectral decomposition reveals multiple frequency components.
- Apocentric libration: Libration occurring near the boundaries of the phase space (0 or $2\pi$), subdivided into clean apocentric libration with clear oscillations despite the boundary effects (Fig. 1(d)), and noisy apocentric libration characteristic of weak resonances in the main asteroid belt where planetary perturbations introduce high-frequency modulations (Fig. 1(e)).

We note that while digital filtering techniques such as the Butterworth filter^12^can potentially reveal the underlying libration in noisy cases, aggressive filtering may introduce false positives under certain conditions, especially for secular resonances^13^, and was therefore not applied in the benchmark construction to maintain methodological rigor.

Non-resonant behavior manifests in several distinct forms requiring separate classification approaches. Circulation occurs when the resonant argument monotonically increases or decreases, producing parallel lines in the time series plot. This category includes:

- Fast circulation: Rapid monotonic change with numerous crossings of the boundaries. The circulation frequency exceeds 10 cycles per integration time (Fig. 1(f)).
- Slow circulation: Gradual change with only a few boundary crossings during the integration period. The circulation frequency is less than 2–3 cycles per integration time (Fig. 1(g)).
- Regular circulation: does not fall into the fast and slow circulations categories.
- Circulation with temporary captures or sticking: Brief episodes of near-resonant behavior (resonance sticking phenomenon), particularly common in regions influenced by both Mars and Jupiter.

Chaotic behavior represents visually a stochastic distribution of points in the resonant argument space (Fig. 1(h)). We distinguish the following classes:

- Pure chaos: Visually unstructured behavior throughout the integration, in which the resonant argument fills the entire vertical range $[0, 2\pi ]$ without clearly visible periodicity or coherent patterns. We stress that the term “chaos” is used here in its colloquial, visual sense to describe the appearance of the time series, not as a formal dynamical characterization.
- Chaos with brief transitions: Short episodes of apparent circulation or even libration that do not persist long enough to change the overall classification.

Transient cases combine elements of both librating and circulating behavior, with clear transitions between these states identifiable through time-frequency analysis (Fig. 1(i)). Some transient cases exhibit additional complexity when planetary perturbations introduce high-frequency noise superimposed on the fundamental dynamical transitions (Fig. 1(j)). Controversial cases represent boundary situations where classification ambiguity arises (e.g., resonant sticking), requiring ensemble averaging over multiple initial conditions to establish confidence in the classification.

### Dataset composition

For this study, we developed several datasets specified below. Their detailed composition is in Table 1.

1. RB–TEST — a test dataset containing eight clear examples that can be used for a quick check of the given model;
2. RB–PILOT — a dataset containing 50 images of different behaviors with simplified outputs (resonant or non-resonant) to compare the results with previous studies;
3. RB–SMALL — a pilot dataset containing 50 images of different behaviors with controversial examples;
4. RB–FULL containing 450 images of all possible behaviors.

The source data (time series of resonant arguments) were taken from numerical simulations performed by the authors for previous studies. The parameters of these simulations varied: different integrators were used, including IAS15(an Everhart-based integrator)^30^, SABA(10,6,4)^31^, WHFast^32^, and SWIFT^9,33^; different dynamical models (all planets; all planets and Pluto); and different time steps and output cadences (ranging from 20 days to 500 yr). Since the benchmark evaluates LLM performance on images of resonant arguments rather than on the underlying numerical data, the specific integration parameters are not critical to the benchmark methodology. This heterogeneity in integration setups also makes the benchmark more representative of real-world usage, where researchers generate resonant argument plots using diverse numerical tools and configurations.

**Table 1:** Composition of the datasets showing the distribution of images across different resonance types and subtypes.

Type	Subtype	TEST	PILOT	SMALL	FULL
Resonant	Pure libration	2	8	3	58
	Slow libration	0	12	3	24
	Noisy libration	0	1	8	13
	Very slow libration	0	0	0	5
	High-amplitude libration	0	0	1	15
	Double libration	0	0	0	11
	Apocentric libration (clean)	1	5	3	17
	Apocentric libration (noisy)	0	0	3	7
Non-resonant	Fast circulation	1	8	5	24
	Slow circulation	1	2	2	26
	Regular circulation	0	0	0	41
	Circulation with sticking	0	0	0	9
	Pure chaos	0	5	5	36
	Noisy circulation	1	0	4	14
Transient	Clean transitions	0	3	5	50
	Noisy transitions	1	0	4	13
	Apocentric transient	1	4	2	57
	Large-amplitude or with a trend	0	3	2	30
Total		8	50	50	450

### Processing workflow

The complete data processing pipeline implemented the following steps. First, resonant argument time series data were collected in CSV format for both mean-motion and secular resonances. The CSV files were then processed through a sophisticated visualization pipeline to generate grayscale scatter plots with consistent formatting: vertical axis ranging from 0 to 2$\pi$ radians, horizontal axis showing the integration timespan, and uniform point size and other settings to ensure standardization across the dataset. The examples of the real images are in Fig. 1 and 3.

Each generated image was submitted to the LLM through its API, OpenRouter service, or local interface, utilizing asynchronous processing, accompanied by the appropriate prompt template optimized for each model category. The LLM response was parsed (from JSON) and then compared against expert labels for metric calculation. This automated pipeline enabled efficient evaluation of multiple models across hundreds of test cases while maintaining consistent experimental conditions, validity, and reliability. The source code and the dataset are available on GitHub.

### Model selection and categories

We evaluated three distinct categories of language models to assess performance across different computational scales and accessibility levels, reflecting the diverse computational resources available to the astronomical community:

- Commercial Large Models: These models, accessible through paid APIs, represent the current state-of-the-art in multimodal AI capabilities. We tested Claude Sonnet 4 (Anthropic), GPT-5 (OpenAI), and Google Gemini 2.5, which offer superior performance metrics but require a subscription.
- Open-Source Large Models: These models provide comparable capabilities to commercial offerings while being freely available under permissive licenses, though they might require (in some cases) substantial computational resources (GPU or RAM) for usage. We evaluated Llama 4 Maverick 17B, Google Gemma 3 27B, Meta Llama 3.2 90B Vision Instruct, and Mistral 3.1 Medium, representing diverse architectural approaches to vision-language understanding.
- Open-Source Small Models: Engineered for deployment on consumer hardware through quantization and architectural optimizations, these models can run on typical workstation computers without specialized GPU infrastructure. We tested Google Gemma 3 4B/12B, Qwen3-vl-8b, Llama 3.2-Vision, and Mistral 3.2 Small, which offer astronomers the ability to perform classification tasks locally without external dependencies.

The distinction between model categories reflects critical practical considerations for astronomical research infrastructure. While large models offer superior accuracy, small models enable researchers to process sensitive or proprietary data locally, avoid API costs for large-scale classification tasks involving millions of objects, and maintain full control over the classification pipeline to ensure reproducibility. Note that for RB-TEST, RB-PILOT, and RB-SMALL we evaluated all selected models, whereas the RB-FULL dataset contains 450 images, making a full benchmark computationally expensive. This research has no financial support and is therefore limited in resources. Consequently, we selected only the best-performing models identified in the previous steps.

### Prompt engineering

Different model categories required distinct prompting strategies optimized to maximize classification performance. For commercial and large open-source models, we developed a comprehensive prompt (see Appendix A) that incorporates detailed analysis steps with hierarchical decision trees, precise percentage thresholds for classification decisions, extensive pattern definitions with visual descriptors validated against expert annotations, and structured JSON output requirements ensuring machine-readable results. Small models, due to their limited context windows, limited computational resources available, and reduced reasoning capabilities, required a simplified prompt focusing on binary classification with basic pattern recognition. The simplified prompt emphasizes visual features directly observable in the plots through simple reasoning, uses plain language descriptions rather than technical terminology to reduce tokenization overhead, and reduces the classification complexity to essential distinctions while maintaining acceptable accuracy.

We note that within each prompt category, all models received exactly the same instructions, ensuring a fair comparison among models of similar capability. The use of two prompt variants (full and simplified) was a practical necessity rather than a methodological choice: pilot experiments showed that small models either fail to follow complex multi-step instructions when given the full prompt. Conversely, large models perform worse with the simplified prompt because it omits the hierarchical decision logic that helps them handle ambiguous cases.

### Evaluation metrics

We employed the classical evaluation strategy to assess model performance across different operational contexts. We calculated standard machine learning metrics: accuracy, precision, recall, and F$\vphantom{0}_1$ Score. We emphasize that these metrics, including the F$\vphantom{0}_1$ score, measure only the performance of the classification of the resonant argument’s behavior, not the physical correctness of resonance identification itself, which can require an extra step as described earlier.

Accuracy measures the overall fraction of correct predictions:

3 $$\begin{aligned} \text {Accuracy} = \frac{\textrm{TP} + \textrm{TN}}{\textrm{TP} + \textrm{TN} + \textrm{FP} + \textrm{FN}} \end{aligned}$$

where $\textrm{TP}$ represents true positives (correctly identified resonant cases), $\textrm{TN}$ represents true negatives (correctly identified non-resonant cases), $\textrm{FP}$ represents false positives (non-resonant cases misclassified as resonant), and $\textrm{FN}$ represents false negatives (resonant cases misclassified as non-resonant).

Precision quantifies the reliability of positive predictions:

4 $$\begin{aligned} \text {Precision} = \frac{\textrm{TP}}{\textrm{TP} + \textrm{FP}} \end{aligned}$$

Recall measures the completeness of positive object predictions, essential for population studies:

5 $$\begin{aligned} \text {Recall} = \frac{\textrm{TP}}{\textrm{TP} + \textrm{FN}} \end{aligned}$$

High recall ensures that few resonant objects are missed, maintaining statistical completeness.

The F$\vphantom{0}_1$ Score provides a balanced measure combining precision and recall through harmonic mean calculation:

6 $$\begin{aligned} \text {F}_1 = 2 \times \frac{\text {Precision} \times \text {Recall}}{\text {Precision} + \text {Recall}} \end{aligned}$$

## Results

Before presenting our results, it is essential to address a fundamental methodological consideration regarding the comparison of classification performance across different studies. Direct comparison of results obtained on different datasets is inherently problematic and potentially misleading. When examining other works dedicated to studying large language models or neural networks for classification tasks, researchers commonly report metrics such as accuracy, precision, recall, F$\vphantom{0}_1$score, and others^2,4,15^, which naturally invites cross-study comparisons. However, such comparisons are methodologically incorrect because the obtained results depend critically on the dataset structure and composition.

This dependency is particularly pronounced in studies of mean-motion resonances, where the vast majority of computed resonant arguments demonstrate non-resonant behavior (either circulation or, more commonly, chaotic dynamics). Consider, for example, a dataset containing resonant arguments from actual asteroid population studies: such a dataset would be significantly imbalanced, with non-resonant cases dominating the sample. Moreover, even basic open-source models would likely demonstrate very good results on such datasets, given that the classification of the simplest cases, such as distinguishing libration from chaotic behavior, represents a simple task that almost all large language models can accomplish with high accuracy. The related high performance would hence reflect the dataset’s inherent bias rather than genuine classification capability on challenging cases.

Therefore, meaningful comparison between different studies is only valid when conducted on identical datasets with consistent labeling criteria and class distributions. This principle guided our benchmark design and underlies our emphasis on stratified sampling rather than naturalistic population statistics.

Table 2 presents the complete performance metrics for all evaluated models across the three benchmark datasets. On the RB-TEST dataset, 10 out of 16 models achieved perfect classification with $100\%$ F$\vphantom{0}_1$ scores. The exceptions were gemini-2.5-flash-lite (F$\vphantom{0}_1$ = $77\%$), gpt-5-nano (F$\vphantom{0}_1$ = $89\%$), grok4-fast (F$\vphantom{0}_1$ = $89\%$; remarkably!), gemma-3-4b-it (F$\vphantom{0}_1$ = $77\%$), mistral-3.2-small-24b (F$\vphantom{0}_1$ = $83\%$), and qwen3-vl-8b (F$\vphantom{0}_1$ = $77\%$). Mostly, the models failed to identify transient cases, classifying them as non-resonant.

**Table 2:** Performance of different LLMs on all datasets. TP, TN, FP, and FN are True Positives, True Negatives, False Positives, and False Negatives, respectively. Acc., Prec., Rec., F$\vphantom{0}_1$ are machine-learning metrics Accuracy, Precision, Recall, and F$\vphantom{0}_1$ score, respectively.

Model	TP	TN	FP	FN	Acc.	Prec.	Rec.	F$\vphantom{0}_1$
**RB-TEST**								
gemini-2.5 pro	5	3	0	0	100%	100%	100%	100%
gemini-2.5-flash	5	3	0	0	100%	100%	100%	100%
gemini-2.5-flash-lite	5	0	3	0	63%	63%	100%	77%
claude-4.5-sonnet-20250805	5	3	0	0	100%	100%	100%	100%
claude-4.5-haiku-20250805	5	3	0	0	100%	100%	100%	100%
gpt-5	5	3	0	0	100%	100%	100%	100%
gpt-5-mini	5	3	0	0	100%	100%	100%	100%
gpt-5-nano	4	3	0	1	88%	100%	80%	89%
grok4-fast	4	3	0	1	88%	100%	80%	89%
llama4-maverick	5	3	0	0	100%	100%	100%	100%
llama-3.2-90b-instruct	5	3	0	0	100%	100%	100%	100%
gemma-3-12b	5	3	0	0	100%	100%	100%	100%
gemma-3-4b-it	5	0	3	0	63%	63%	100%	77%
mistral-3.2-small-24b	5	1	2	0	75%	71%	100%	83%
mistral-medium-3.1	5	3	0	0	100%	100%	100%	100%
qwen3-vl-8b	5	0	3	0	63%	63%	100%	77%
**RB-PILOT**								
**gemini-2.5 pro**	33	17	0	0	100%	100%	100%	**100%**
gemini-2.5-flash	33	16	1	0	98%	97%	100%	99%
gemini-2.5-flash-lite	33	5	12	0	76%	73%	100%	85%
**claude-4.5-sonnet-20250805**	33	17	0	0	100%	100%	100%	**100%**
claude-4.5-haiku-20250805	33	16	1	0	98%	97%	100%	99%
**gpt-5**	33	17	0	0	100%	100%	100%	**100%**
gpt-5-mini	33	16	1	0	98%	97%	100%	99%
gpt-5-nano	31	16	1	2	94%	97%	94%	95%
grok4-fast	33	16	1	0	98%	97%	100%	99%
llama4-maverick	33	4	13	0	74%	72%	100%	84%
**llama-3.2-90b-instruct**	32	14	3	1	92%	91%	97%	**94%**
**gemma-3-12b**	32	14	3	1	92%	91%	97%	**94%**
gemma-3-4b-it	33	0	17	0	66%	66%	100%	80%
mistral-3.2-small-24b	32	2	15	1	68%	68%	97%	80%
**mistral-medium-3.1**	33	15	2	0	96%	94%	100%	**97%**
qwen3-vl-8b	33	5	12	0	76%	73%	100%	85%
**RB-SMALL**								
**gemini-2.5 pro**	32	14	2	2	92%	94%	94%	**94%**
gemini-2.5-flash	23	16	0	11	78%	100%	68%	81%
gemini-2.5-flash-lite	20	12	4	14	64%	83%	59%	69%
claude-4.5-sonnet-20250805	27	16	2	5	86%	93%	84%	89%
claude-4.5-haiku-20250805	19	16	2	13	70%	91%	59%	72%
gpt-5	22	16	0	12	76%	100%	65%	79%
gpt-5-mini	28	15	2	5	86%	93%	85%	89%
gpt-5-nano	19	15	2	12	71%	91%	61%	73%
grok4-fast	15	16	0	19	62%	100%	44%	61%
llama4-maverick	20	7	9	14	54%	69%	59%	64%
**llama-3.2-90b-instruct**	21	16	3	10	74%	88%	68%	**76%**
gemma-3-12b	12	16	0	22	56%	100%	35%	52%
gemma-3-4b-it	18	14	3	15	64%	86%	55%	67%
mistral-3.2-small-24b	24	1	16	9	50%	60%	73%	66%
mistral-medium-3.1	16	16	3	15	64%	84%	52%	64%
qwen3-vl-8b	14	14	2	20	56%	88%	41%	56%
**RB-FULL**								
gemini-2.5 flash	177	147	17	109	72%	91%	62%	74%
claude-4.5-sonnet-20250805	144	147	27	132	65%	84%	52%	64%
gpt-5-mini	183	143	21	102	73%	90%	64%	75%
**RB-FULL SIMPLIFIED**								
gemini-2.5 flash	285	129	21	15	92%	93%	95%	94%
claude-4.5-sonnet-20250805	274	132	18	26	90%	94%	91%	93%
**gpt-5-mini**	289	138	12	11	95%	96%	96%	**96%**
**llama-3.2-90b-instruct**	290	111	37	10	90%	89%	97%	**93%**
gemma-3-12b	260	125	25	40	86%	91%	87%	89%

On the RB-PILOT dataset, three commercial flagship models achieved perfect performance: gemini-2.5-pro, claude-4.5-sonnet-20250805, and gpt-5, all with $100\%$ F$\vphantom{0}_1$ scores. Four additional commercial models achieved $99\%$ F$\vphantom{0}_1$ scores with single classification errors: gpt-5-mini, grok4-fast, gemini-2.5-flash, and claude-4.5-haiku-20250805. Each of these models made errors on different examples within the dataset. Among open-source models, mistral-medium-3.1 achieved the highest F$\vphantom{0}_1$ score of $97\%$, followed by llama-3.2-90b-instruct and gemma-3-12b, both at $94\%$. The lowest performing models were gemma-3-4b-it (F$\vphantom{0}_1$ = $80\%$) and mistral-3.2-small-24b (F$\vphantom{0}_1$ = $80\%$).

The RB-SMALL dataset, which required three-way classification (resonant, non-resonant, transient) and contained a lot of unclear and highly controversial cases, produced substantially lower performance metrics across all models: gemini-2.5-pro achieved the highest F$\vphantom{0}_1$ score of $94\%$, followed by claude-4.5-sonnet-20250805 and gpt-5-mini, both at $89\%$. The model gpt-5 (remarkably) achieved $79\%$ F$\vphantom{0}_1$ score, lower than its smaller variant gpt-5-mini. Among open-source models, llama-3.2-90b-instruct led with $76\%$ F$\vphantom{0}_1$ score, while other open-source models ranged from $52\%$ to $67\%$. gemma-3-12b, which achieved $94\%$ on RB-PILOT, dropped to $52\%$ F$\vphantom{0}_1$ score on RB-SMALL.

On the RB-FULL dataset, we ran the full benchmark for three models: gemini-2.5-flash, claude-4.5-sonnet-20250805, and gpt-5-mini, and a simplified version (without the transient class) for gemini-2.5-flash, claude-4.5-sonnet-20250805, gpt-5-mini, llama-3.2-90b-instruct, and gemma-3-4b-it. In the full benchmark, gemini-2.5-flash and gpt-5-mini achieved the best performance (F$\vphantom{0}_1\approx 75\%$). In the simplified version, the open-source LLMs llama-3.2-90b-instruct and gemma-3-12b-it reached comparable performance ($93\%$ and $89\%$, respectively, vs. an average of $94\%$ for commercial models). Most misclassifications correspond to circulation with sticking cases and chaotic behavior with short episodes of libration or circulation.

All models within each category (large and small) used identical prompts to ensure fair comparison, although it is possible (and verified) that prompt engineering per LLM can improve performance significantly. The prompts were written by the first author, incorporating astronomical domain knowledge rather than being optimized for individual model architectures, and they have also been reviewed by the second author. For RB-SMALL, experiments during prompt engineering showed that model-specific optimization could improve individual model performance but at the cost of decreased performance on other models.

## Discussion and conclusion

The results reveal several important patterns in model behavior across different complexity levels. The near-universal perfect performance on RB-TEST demonstrates that modern LLMs can effectively handle straightforward resonance classification tasks. The systematic failure of some models to identify transient cases, despite correctly classifying pure resonant and non-resonant examples, suggests architectural limitations in detecting complex visual patterns with a non-fine-tuned prompt. The lower performance of small models on this dataset is partially attributable to the limited sample size, where single errors substantially impact percentage-based metrics.

The RB-PILOT results demonstrate a clear performance hierarchy among model categories. Flagship commercial models consistently achieve near-perfect accuracy, validating their position as state-of-the-art solutions. The observation that different models made errors on different examples has significant practical implications: ensemble approaches combining multiple mid-tier models could potentially achieve flagship-level accuracy at substantially reduced computational cost because often, different models misclassified different images. The strong performance of gemma-3-12b ($94\%$ F$\vphantom{0}_1$), a model capable of running on standard workstation hardware, is particularly significant for democratizing access to high-quality astronomical classification tools.

The significant performance reduction on RB-SMALL across all models illuminates the challenge of complex astronomical classification. The dataset’s inclusion of resonance sticking events, temporary trapping phenomena, and motion near separatrixes represents cases where even human experts may disagree. The requirement for three-way classification rather than binary categorization (like it was in previous studies)^2,4,15^ substantially increased task complexity, as models must distinguish not only between resonant and non-resonant behavior but also identify transitional states that may persist for varying durations.

The counterintuitive superior performance of gpt-5-mini over gpt-5 on RB-SMALL suggests that model complexity does not monotonically correlate with classification accuracy for specialized visual tasks. Several hypotheses may explain this phenomenon: smaller models may be less prone to overthinking complex patterns, simplified architectures might better match the pattern recognition requirements of the task, or training regimes for smaller models may have emphasized practical classification over abstract reasoning capabilities. This finding suggests that the optimal model for a given classification task may not simply be the largest or most sophisticated available and therefore has to be studied in advance by researchers.

The substantial performance gap between commercial and open-source models on RB-SMALL ($94\%$ versus $76\%$ maximum F$\vphantom{0}_1$ scores) primarily manifests in cases requiring distinction between transient and non-resonant behaviors. These cases often involve high-frequency oscillations or temporary resonance captures where subtle temporal patterns determine correct classification. The difficulty lies not merely in pattern recognition but in understanding temporal evolution and identifying transition points between dynamical regimes.

It should be noted that the “transient” category, as defined in the present benchmark, includes several dynamically distinct phenomena, including temporary resonance captures, resonance sticking, separatrix-crossing or near-separatrix behavior, and other borderline regimes. In principle, a finer classification scheme is desirable, which is (theoretically) possible. For example, a temporary capture is expected to exhibit libration of the resonant argument at frequencies close to the frequencies of oscillations of the semi-major axis, persisting over a time interval sufficient for reliable identification and having a small number of revolutions (cycles) of the unwrapped resonant angle. Resonance sticking, by contrast, may manifest as a slow linear drift of the resonant argument with (false) librations^9^. However, such distinctions are straightforward only in idealized dynamical models. In real cases, especially for high-order mean-motion resonances in the main asteroid belt^34^, the resonant argument is significantly contaminated by planetary perturbations, and even phase-portrait analysis does not always provide unambiguous discrimination between these regimes.

Constructing a benchmark with physically motivated transient subcategories would therefore require detailed dynamical investigation of each individual case, which is prohibitively time-consuming at the population scale. The pragmatic approach adopted here is to separate clearly resonant and clearly non-resonant cases (which can also be handled by standard algorithmic criteria with high accuracy) and to flag all other cases as transient, prioritizing high recall so that no dynamically interesting object is missed. The resulting transient class can subsequently be refined by the researcher into physically motivated subcategories. We emphasize that even with this three-class scheme, the LLM-based approach produces substantially fewer false positives in ambiguous regimes than traditional deterministic algorithms, particularly for high-order resonances, and requires less time and effort to complete the resonant angle’s classification step within the identification pipeline^21,34^.

We also note that no systematic difference in model performance was observed between mean-motion and secular resonances. Although the generated images depend on technical parameters such as the integration timespan, output step, and the resulting visual density of points, the underlying dynamical distinction between these two classes of resonances does not appear to affect LLM classification accuracy. This suggests that the models respond primarily to the geometric structure of the resonant argument time series (bounded oscillations versus unbounded drift or stochastic scatter) rather than to features specific to a particular resonance type, which is consistent with the zero-shot, domain-agnostic nature of the approach.

Our results suggest that complex classification instructions might benefit from hierarchical decomposition. A two-stage approach, where the first stage separates resonant from non-resonant cases and the second stage identifies transient behavior within the resonant category, could potentially improve accuracy while reducing computational requirements. This strategy mimics human reasoning (split a task into smaller sub-tasks) and could be implemented through structured prompt engineering or explicit multi-stage processing pipelines.

Comparing our LLM-based approach with traditional methods reveals distinct advantages. Numerical methods such as frequency analysis require substantial coding and expert analysis. Machine learning approaches using neural networks or classical machine learning require extensive labeled training sets, adaptation for the given task, and, what is more important, they struggle with edge cases. LLMs combine the advantages of both paradigms: like classical methods, they require no training on specific datasets, and like machine learning methods, they do not require researchers to spend a lot of time on coding and testing.

Overall, the developed benchmarks and the obtained results clearly demonstrate that both commercial and open-source LLMs can accurately classify resonant arguments for both mean-motion and secular resonances. Even small models, such as gemma-12b-it, which one can run on one’s personal computer, achieve reasonable performance on complex cases. For clear images and the two-category framework, many models exhibit near-perfect accuracy, validating their applicability in real-life scenarios. The present study also provides a standardized approach for testing new models on unified benchmarks, which can be used by other researchers to validate their results.

## Data Availability

All data used is available for review and use. The datasets are available on Zenodo: https://doi.org/10.5281/zenodo.17709163. The code used to obtain the benchmark results is available on github: https://github.com/smirik/llm-libration.

## Appendix A: Prompt Templates

### Prompt for the full classification

PROMPT_TEMPLATE=”You are an expert astronomer specializing in the analysis of orbital dynamics from scatter plots. Your task is to classify a given plot of a resonant angle versus time into one of three categories: resonant, non-resonant, transient.\n\nresonant: the data points are confined within clear horizontal boundaries for the entire duration of the plot. The plot can be within these boundaries OR it can be split into two blocks connected to the top and bottom borders, where each block stays within its own boundaries. In this case, one can draw a rectangle in the vertical middle of the plot that has NO dots in it. If there are no such boundaries, the plot is NOT resonant.\n\nnon-resonant: chaotic scatter that fills most or all of the vertical space OR clear diagonal lines that \”wrap around\”from the top of the plot to the bottom. The lines can be noisy or have some oscillations, but the general trend is parallel diagonal lines. If this behavior lasts ALL the plot, then it is non-resonant.\n\ntransient: if the plot does not fall in resonant or non-resonant, then it’s transient. Transient means that there is a piece of the plot that behaves as resonant, but not all the time.\n\nNote: some plots can be noisy and have several \”trends\”or mixed behaviors. In this case, it’s better to classify them as transient.\n\nTo perform the classification, follow the steps:\n1.Split image into blocks 10% each.\n2.Classify each block.\n3.If each block is resonant, the status is resonant.\n4.If each block is non-resonant, the status is non-resonant.\n5.In all other cases, it is transient.\n\nClassify the image in one of these categories following the algorithm above.”

### Prompt for the simplified classification

SIMPLE_PROMPT_TEMPLATE=”You are an expert astronomer specializing in the analysis of orbital dynamics from scatter plots. Your task is to classify a given plot of a resonant angle versus time into one of two categories: resonant, non-resonant. Review it as an image. Rules: non-resonant: when there is chaotic scatter that fills ALL of the vertical space OR clear diagonal lines that wrap around from the top of the plot to the bottom. The lines can be noisy or have some small oscillations, but the general trend is parallel diagonal lines or random scatter across the ENTIRE plot. There should be no oscillations (even short ones) or two blocks of oscillations near the borders. resonant: if there are some oscillations OR a part of them, the status is ALWAYS resonant. Important: you must review the WHOLE graph, not a part of it.”
